# Examining the Link Between Social Affect and Visual Exploration of Cute Stimuli in Autistic Children

**DOI:** 10.1007/s10803-024-06504-1

**Published:** 2024-08-22

**Authors:** Alexandra Zaharia, Nada Kojovic, Tara Rojanawisut, David Sander, Marie Schaer, Andrea C. Samson

**Affiliations:** 1 Faculty of Psychology, UniDistance Suisse, Brig, Switzerland; 2 Institute of Special Education, University of Fribourg, Fribourg, Switzerland; 3 Swiss Center for Affective Sciences, University of Geneva, Geneva, Switzerland; 4 Department of Psychiatry, Faculty of Medicine, University of Geneva, Geneva, Switzerland; 5 Faculty of Sciences, University of Geneva, Geneva, Switzerland; 6 Faculty of Psychology and Educational Sciences, University of Geneva, Geneva, Switzerland; 7 Fondation Pôle Autisme, Unité de Recherche, Geneva, Switzerland

**Keywords:** Social affect, Autism spectrum disorder, Cuteness, Baby schema, Eye-tracking

## Abstract

*Baby schema* refers to physical features perceived as cute, known to trigger attention, induce positive emotions, and prompt social interactions. Given the reduced visual attention to social stimuli observed in individuals on the autism spectrum, the current study examines whether the sensitivity to baby schema is also affected. We expected that the looking time towards cute-featured stimuli would vary with symptom severity levels and would be associated with social affect. Ninety-four children (31 typically developing; 63 diagnosed with autism spectrum disorder - ASD) aged 20–83 months (*M* = 49.63, *SD* = 13.59) completed an eye-tracking visual exploration task. Autistic participants were separated into two groups based on symptom severity: children with high autism severity symptoms (HS ASD; *N* = 23) and low-moderate autism symptoms (LMS ASD; *N* = 40). Animals and neutral objects were simultaneously presented on the screen along with either human babies (condition 1) or adults (condition 2). The results indicated that visual attention oriented to cute-featured stimuli varied with autism symptom severity: only LMS and TD groups spend more time looking at cute-featured stimuli (*babies*; *animals*) than *neutral objects*. Moreover, children with higher severity in the social affect domain spent less time on the stimuli depicting cute than non-cute stimuli. These findings suggest that autism symptom severity and social skills are linked to variations in visual attention to cute stimuli. Implications of baby schema sensitivity are discussed in relation to the development of social competencies and play, responsiveness to robot-based interventions, as well as appraised relevance in autistic children.

**Supplementary Information:**

The online version contains supplementary material available at 10.1007/s10803-024-06504-1.

The baby schema (*Kindchenschema*) refers to a set of features described as cute (round head, big eyes, chubby cheeks, etc.) that naturally triggers interest and attention, caretaking and protection behavior (Lorenz, 1943; Yao et al., 2022). This universal and cross-cultural response to the baby schema is known as the cuteness effect (Lorenz, 1943; Luo et al., 2015; Yao et al., 2022). Cute-featured stimuli, commonly babies and animals, have high emotional content, elicit positive emotions, release oxytocin, decrease anxiety, and promote bonding, social interactions, empathy, prosocial, and play behavior (Borgi et al., 2014; Doebel et al., 2022; Golonka et al., 2023; Levinson, 2022; Takamatsu, 2023; Yao et al., 2022). They also activate dopaminergic reward systems and brain regions related to motor approach behavior, attachment, emotion processing, and theory of mind (Luo et al., 2015). From an evolutionary perspective, survival of vulnerable youngsters may depend on rapidly attracting the attention of adults (Brosch et al., 2007, 2008; Glocker et al., 2009; Lei et al., 2020), but also of other children (Glocker et al., 2009; Saxton et al., 2020). The limited research in children shows that the cuteness response emerges already around the age of two (Borgi & Cirulli, 2013; Doebel et al., 2022; Saxton et al., 2020), and that the baby schema impacts gaze allocation in 3–6 years old children (Borgi et al., 2014). One study indicated that 3- to 12-month-old infants with no sibling or nursery experience show a weak visual preference bias towards infant faces compared to child faces (Damon et al., 2021). Yet, due to the lack of strong evidence, it is unclear whether sensitivity to baby schema can emerge in young toddlers. Finally, sensitivity to baby schema nurtures relationships (e.g., bonding, affiliation) and represents an early marker of social skills.

Exploring the sensitivity to baby schema in children presenting socio-emotional difficulties, such as those on the autism spectrum, could shed light on early processes that potentially impede engagement in social interactions, motivation, and interest (Cai et al., 2019; Hadjikhani et al., 2014; O’Connor et al., 2019). An atypical perception of environmental cues has been linked to reduced helping behavior in autistic individuals (Komeda et al., 2019). Identifying whether atypical patterns extend to the baby schema could provide insight into the underlying mechanisms of social behavior and appraised relevance. For instance, the nature of attention patterns toward baby schema may indicate alterations in social reward systems or in appraisals of socio-emotional triggers that support bonding, caregiving, and positive relations.

Many studies, mostly using eye-tracking, generally indicate attenuated attention toward social stimuli during visual exploration in autistic individuals (Bast et al., 2021; Chita-Tegmark, 2016; Frazier et al., 2017). Although some studies showed that autistic children are more likely to direct their attention to non-social stimuli that represent circumscribed interests than to social stimuli (i.e., children and adults expressing happy faces in Sasson, Dichter et al., 2012; i.e., emotional faces of adults in Sasson & Touchstone, 2014), others found no differences between autistic and typically developing (TD) children in the time spent on social vs. non-social stimuli (Chita-Tegmark, 2016). These mixed results may be linked to task-related characteristics (e.g., type or quantity of social stimuli, type of interactive agent) and participant-related (e.g., profile heterogeneity, social skills, autism symptom severity) (Bast et al., 2021; Chawarska et al., 2016), and impact potential intervention outcomes. Indeed, the literature seems to suggest that the variations observed in the visual exploration patterns and preferences of social stimuli are often linked to the participants’ social skills level. For example, autistic children’s socialization and communication skills were positively linked to the time spent on dynamic social stimuli depicting children (Franchini et al., 2017). Also, the children’s overall preference for social stimuli (i.e., animals and human beings) was negatively associated with autism symptom severity (Celani, 2002). Interestingly, the performance in processing adult faces was found to be more strongly linked to autism symptom severity in the social affect domain than in the repetitive and restricted behavior (RRB) domain (Zagury-Orly et al., 2022).

Regarding attention to cute stimuli in autistic individuals, the evidence is limited. To the best of our knowledge, the only study addressing cuteness sensitivity in autism is an unpublished work (Sasson et al., 2012), in which a behavioral task was administered in a small sample of autistic adults (*N* = 9). The researchers observed that autistic participants perceived less the infant cuteness compared to the TD group, which would suggest the presence of an altered social reward processing. Another study showed that children with lower autism symptoms preferred robots presenting cute features, whereas children with more severe symptoms preferred humanoid-like robots resembling adults (Kumazaki et al., 2017). In a preference-based task (Prothmann et al., 2009), autistic children preferred interacting with dogs, seconded by persons (adults) and then by objects, but the small sample had a large range in intellectual capacities and symptom severity was not controlled. Another study found no differences in the preference for animals vs. inanimate objects in autistic children (Celani, 2002). A recent literature review suggested that, when looking at animals, autistic children’s eye gaze patterns are comparable to those with TD, but their visual attention is more biased toward animals than humans (Toutain et al., 2024). Nonetheless, a notable limitation in the findings presented in this article lies in the infrequent reporting of symptom severity across the referenced studies.

Despite these different results, autism profile heterogeneity should be considered when examining social attention. Sensitivity to social stimuli might be linked to symptom severity, particularly, by impairments in social skills (Franchini et al., 2017). The relationship between the social affect and RRB domains in autistic children is still unclear (Chaxiong et al., 2022). It is considered that social affect and RRB contribute independently to the autism diagnosis, present distinct developmental trajectories, and impact differentially the responsiveness to interventions (Gotham et al., 2007; Hus et al., 2014). Given their different nature and contributions to autistic behaviors and profiles, treating them separately might help to unmask the specific effects and weight that each may have on the visual processing of cute stimuli. Finally, examining visual processing of cute-featured stimuli in groups with varying severity of autism symptoms may help understand how attentional mechanisms affect social skills development.

These overreaching observations point towards an altered perception and processing of social, and perhaps cute, cues in autism, that may appear very early in development (e.g., infancy, see Chita-Tegmark, 2016). The evidence hints at the possibility that cute-featured objects may trigger distinct socio-affective responses, depending on the participants’ social skill levels. These responses could be discerned through patterns of visual attention and preference. We then may expect that autistic children exhibit distinct visual patterns when exploring cute stimuli (i.e., human babies and non-human animals) in comparison to their TD peers. Yet, the degree of dissimilarity from their TD peers may fluctuate, as indicated by existing literature, with the autism symptom severity levels, particularly within the domain of social skills. Thus, the current study aimed at examining the attention to stimuli with baby schema features (i.e., [human] babies and [non-human] animals) in autistic children with varying symptom severity (low-moderate versus high) compared to TD children. A novel eye-tracking paradigm was proposed: a brief visual exploration task including social stimuli with cute features (human babies, non-human animals) and without cute features (human adults), and inanimate non-social stimuli (neutral objects). Given the few and ambiguous findings regarding the precise age at which the cuteness response emerges, we expanded the scope of this new study to examine participants with ages between 1 and 6 years old. The study pursued a twofold goal.

First, we investigated the link between the visual patterns of cute stimuli and autism symptoms. We expected that the attentional bias towards cuteness-configured stimuli would vary across groups and depend on the autism symptom severity. Consequently, we assessed the percentage of looking time spent (i.e., *fixation percentage)* on the areas of interest (AOIs), a widely-used eye-tracking parameter (Chita-Tegmark, 2016). We hypothesized that only children with lower symptom severity and TD peers would allocate more time to cute social stimuli (animals, babies), compared to non-cute social stimuli (adults and/or neutral objects). We expected then that the visual attention bias towards cute stimuli is reduced in children with higher autistic symptoms compared to the other two groups. We also explored the engagement with the stimuli (i.e., *average fixation duration*) as well as the initial orientation (i.e., *time to first fixation*) on AOIs.

Second, we examined the link between the sensitivity to cuteness, further labeled as the baby sensitivity index (percentage of looking time spent on all “cute” stimuli versus all “non-cute” stimuli), and symptom severity in the two core domains described in the autism spectrum disorder (ASD) diagnosis (i.e., social affect and RRB). A second baby sensitivity index was calculated specifically on human stimuli (percentage of looking time spent on babies as “cute” humans versus adults as “non-cute” humans) and tested for links with the symptom severity in the social affect and RRB. We expected that increased attention towards cute over non-cute stimuli and babies over adults, respectively, is linked to social affect only.

## Methods

### Participants

Participants included in the current study are part of the a longitudinal autism cohort, that started in 2012 (Geneva Autism Cohort), in which children are invited for assessments every six months for two years, described elsewhere (Franchini et al., 2017; Robain et al., 2020). Children on the autism spectrum were recruited through French and English-speaking parent associations and clinical centers. TD peers were recruited through local announcements and word-of-mouth. Parents or legal guardians gave their written informed consent for participation. The study was approved by the local ethics committee.

Inclusion criterion for all participants was an age of 1 to 6 years. Moreover, the autistic children were required to satisfy DSM 5 criteria for Autism Spectrum Disorder (ASD; American Psychiatric Association, 2013), and have their clinical diagnosis confirmed using the ADOS (Autism Diagnostic Observation Schedule-Generic; ADOS-G; Lord et al., 2000, Autism Diagnostic Observation schedule, second edition; ADOS-2; 2012; described in the section below). Finally, the TD children were screened for any developmental concerns, autism symptoms (see Table 1), and/or history of ASD in first-degree relatives.

**Table 1:** Participants’ socio-demographic characteristics and scores (mean and standard deviation) on autism symptoms, adaptive behavior and cognitive skills

	HS ASD	LMS ASD	TD
N total	23	40	31
Age (months)			
Mean (*SD*)	45.86 (9.93)	48.93 (12.24)	53.31 (16.73)
Age range	24–59	24–70	20–83
Gender ratio (male/female)	18/5	34/6	21/10
Parents’ education (N and %)			
Primary/Elementary school	1 (4.30%)	2 (5%)	0 (0%)
High school	6 (26.10%)	4 (10%)	3 (9.70%)
University	14 (60.90%)	34 (85%)	28 (90.30%)
NA	2 (8.70%)	0 (0%)	0 (0%)
Autistic symptoms (ADOS; Mean and *SD*)			
Total CSS	8.74 (0.69)	5.98 (0.95)	1.07 (0.26)
Total CSS Range	8–10	4–7	1–2
Social Affect CSS	7.43 (1.44)	4.70 (1.29)	1.07 (0.26)
RRB CSS	9.39 (1.44)	8.88 (1.24)	2.10 (1.86)
Adaptive behavior (VABS-II; Mean and *SD*)			
Adaptive Behavior	82.48 (12.39)	83.16 (10.10)	101.77 (9.76)
Communication	84.74 (19.29)	85.46 (12.36)	104.77 (10.18)
Daily Living Skills	84.52 (10.75)	86.76 (10.63)	99.46 (8.08)
Socialization	80.83 (9.41)	81.62 (10.10)	97.15 (8.95)
Motor Skills	88.96 (12.31)	89.43 (10.79)	104.24 (15.00)
Cognitive skills (MSEL; Mean and *SD*)			
Total	74.65 (24.41)	77.59 (21.45)	115.81 (14.88)

*Note* Missing data: in the LMS ASD group: VABS-II *N* = 3, MSEL *N* = 1; in the TD group: VABS-II *N* = 5, MSEL *N* = 4, ADOS *N* = 1. Abbreviations: RRB = Repetitive and restrictive behaviors, CSS = Calibrated severity score, HS = High severity autism spectrum disorder, LMS = Low-moderate severity autism spectrum disorder, TD = Typically developing, ADOS = Autism Diagnostic Observation schedule, VABS-II = Vineland Adaptive Behavior Schedule-second edition, MSEL = Mullen Scales of Early Learning, NA = no answer

A total of 164 children completed the eye-tracking task. Seventy children (42%) were excluded for attending each of the six frames presented during the visual exploration eye-tracking task for less than 50% of the exposition time. The threshold was chosen based on previous eye-tracking data preparation used in research with autistic toddlers (Pierce et al., 2011, 2016). To our knowledge, no compromise exists yet regarding a viewing time threshold in research on autistic children. The final sample retained for analyses included 63 autistic and 31 TD children.

Based on the overall ADOS total calibrated severity score (CSS) and the CSS classification and cutoffs indicated in the ADOS-2 manual (Gotham et al., 2009; Hus et al., 2014; Lord et al., 2012), two ASD subgroups were distinguished within the autistic group. The first subgroup included participants with high severity autism symptoms (HS ASD; N = 23) who have obtained a total severity score ranging from 8 to 10. The second subgroup (i.e., participants having low to moderate autism symptoms; LMS ASD) included participants with scores indicating low (score ranges 3–4; N = 5), and moderate severity autism symptoms (score range 5–7; N = 35). The two groups (HS ASD and LMS ASD) were significantly different only on the ADOS social affect CSS (*t*(61) = 7.80, *p* < .001). There were no other significant differences (*p* > .05) between the two ASD groups regarding measures of RRB CSS, adaptive behavior (Vineland Adaptive Behavior Schedule-second edition; VABS-II; Sparrow et al., 2005), nor cognitive skills (Mullen Scales of Early Learning; MSEL; Mullen, 1995) (See Table 1). The three groups (HS ASD, LMS ASD, and TD) did not significantly differ in gender (χ^2^ = 3.01, *p* = .22) or age (*F*(2,94) = 1.76, *p* = .18).

For a description of participants’ socio-demographic characteristics (age, gender, and parents’ education level), and scores on autism symptoms, adaptive behavior, and cognitive skills, see Table 1. Following previous research (Wood de Wilde et al., 2023), we used the parents’ educational level (highest educational level attained among the two parents) to depict the socio-economic background of our participants. The educational level of parents was summarized along three categories: elementary/primary school, high school, and university. Parents were also asked to provide information regarding their nationality(ies) and the language(s) spoken to the child at home. The present sample illustrates the multicultural diversity of the recruited participants: 34 different nationalities and 17 different spoken languages, which is representative of the geographic region in which the study took place (see Wood de Wilde et al., 2023). Among the 34 nationalities, 13 are European and the 21 left are African, North and South American, Asian, and Australian.

### Measures

#### Autism Symptoms

The ADOS is a semi-structured assessment measuring the overall symptomatology levels and the presence of autism symptoms across two domains: social affect and RRB (Lord et al., 2000, 2012). In general, social affect items refer to communication and reciprocal social interaction skills. RRB items refer, for example, to stereotyped/idiosyncratic use of words/phrases, unusual sensory interest, mannerisms, and excessive interest in specific topics. To facilitate the comparison of scores from ADOS-G and ADOS-2 versions, the raw scores were transformed into standardized CSS according to previously published procedures (Esler et al., 2015; Gotham et al., 2007, 2009; Hus et al., 2014). Three scores were obtained: an overall total CSS, the social affect CSS, and the RRB CSS. The CSS ranges on a 10-point scale. Based on the theoretical cutoffs and labels from ADOS-2 manual instructions (Lord et al., 2012), a quantitative assessment of autism symptom severity using the CSS classified participants’ total scores as following: 1–2 as minimal to no evidence, 3–4 as low severity, 5–7 as moderate severity and 8–10 as high severity.

#### Adaptive Behavior

Children’s adaptive behavior was assessed using the VABS-II (Sparrow et al., 2005), which is a semi-structured standardized parent report interview. Standardized scores were obtained for the following domains: Communication, Daily Living, Socialization Skills, and Motor Skills. An Adaptive Behavior composite score was calculated to obtain an overall measure of adaptive functioning.

#### Cognitive Skills

Participants’ developmental levels were measured using MSEL (Mullen, 1995). An early learning composite score (M = 100, SD = 15) was calculated based on four cognitive domains: fine motor, visual reception, receptive language, expressive language scales. The score provides a measure of general cognitive functioning.

### Stimuli and Procedure

Our novel eye-tracking Visual Exploration Task (VET) and procedure were inspired by Sasson and colleagues (2008). First, 120 colored static stimuli (i.e., 15 human adults, 15 human babies, 30 animals, and 60 neutral objects) were selected using several sources. The human stimuli were a mix of faces and the upper body of people. Baby faces were selected from the freely available copyright protected *Children Affective Facial Expression* set (LoBue, 2014; LoBue & Thrasher, 2015). Adult faces were selected from the freely available copyright protected resource of *Chicago Faces Database* (Ma et al., 2015). All human stimuli were depicting neutral faces (to exclude any potential emotional attention biases). The animals (cats and dogs only) and neutral objects were retrieved from Google search engine. Age of animals was not controlled. The neutral objects were selected from domains that are not known to represent common circumscribed interest of individuals on the autism spectrum (i.e., these objects are referred to as low autism-interest stimuli such as furniture, bathroom and kitchen objects, office supplies, etc.; see Sasson et al., 2008).

Second, to examine the emotional content of the stimuli (see Pool et al., 2016) for the study goal, forty-four adults aged 21–49 years (27 females, 15 males; *M*_age_ = 29.19, *SD*_age_ = 9.37) have rated the selected images in terms of cuteness and pleasantness, on a 5-point Likert scale (1= “not cute at all”/ “very unpleasant” and 5= “very cute”/ “very pleasant”) in an online survey. When comparing stimuli depicting *animals* to *human babies*, there were no significant differences in terms of cuteness, nor valence. *Animals* and *human babies* were rated significantly cuter and more pleasant than *adults* and *neutral objects* (*p* < .001). *Adults* were rated significantly cuter and more pleasant than *neutral objects* (*p* < .001). Therefore, the emotional content was found higher in stimuli depicting *babies* and *animals* compared to *adults* and *neutral objects*, as well as in stimuli depicting *adults* compared to *neutral objects* (see Table 2). All rated stimuli were retained for the task.

**Table 2:** Means (*M*) and standard deviations (*SD*) for the cuteness and pleasantness ratings for each stimuli category

Stimulus category	Cuteness	Pleasantness
	*M* (*SD*)	*M* (*SD*)
Human adults	3.43 (0.29)	3.43 (0.29)
Human babies	3.76 (0.38)	3.40 (0.29)
Animals	3.74 (0.37)	4.12 (0.22)
Neutral objects	1.56 (0.29)	2.95 (0.38)

Next, the 120 stimuli were randomly distributed on six frames on a white background. Each frame contained 20 visual stimuli from the three different categories: ten neutral objects, five animals, and five humans (human adults in Condition 1 and human babies in Condition 2). Although stimuli originated from several sources, luminosity differences between stimuli were expected to have a low impact on attention given the simultaneous presentation of twenty stimuli on the screen. To control for perceptually driven attention biases, patterns on clothes were avoided and saturation was harmonized. Human gender (female, male), ethnicities, animal species (cats, dogs), the type of neutral objects, and the form of stimuli depicting beings (standing persons, face only) were balanced across frames.

**Fig. 1:**
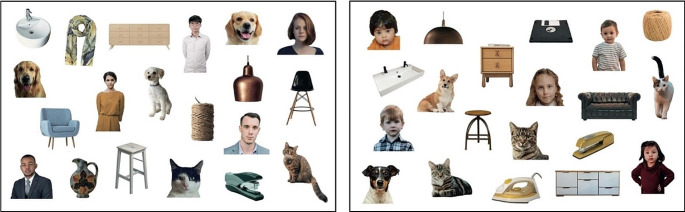
The left image represents an example of a frame adults-animals-neutral (condition 1). The right image represents an example of a frame babies-animals-neutral (condition 2). *Note*: This is a reconstruction with freely available images with no copyright restrictions, as the images used in the original task are partially copyright protected

Half of the frames (three out of a total of six) included human adult stimuli (condition 1), while the other half included human baby stimuli (condition 2) (see reconstruction of frames with freely available images in Fig. 1). To avoid biases and increase randomization, two versions of the VET were created (A and B). The second version (B) was built using the same 120 stimuli from version A and six new frames were built by obtaining new combinations of the stimuli. The stimuli ratio on each frame was maintained, but their positions on the frame and across the frames were changed.

The six frames were presented randomly for 10 s each (the total duration of each 3-frame condition was 30 s). Each frame was preceded by a 500-millisecond fixation target. Overall, the VET lasted 63 s.

The task was implemented in Tobii Studio Software, version 3.2.3, and administered on a Tobii TX300 eye-tracker (Tobii, Sweden, www.tobii.com*)* with 23-inch display, 1920 × 1200 resolution (72 dpi), and a sampling rate of 300 Hz. Participants were positioned at approximately 60 cm from the screen. Raw data were filtered using the I-VT filter (default parameters, minimal fixation duration threshold set at 60 milliseconds) (Olsen, 2012). A nine-point calibration procedure was completed before showing the stimuli. To ensure a satisfactory eye gaze data collection, the calibration was performed in operator paced-mode. If any of the 9 calibration points was missed or showed error vectors, the calibration was repeated. The task was administered uniquely if all the calibration points were satisfactory (no error vectors). In each of the two conditions, the stimuli were divided in three AOIs: humans, animals, and neutral objects.

Participants were randomly assigned to version A or B and instructed to freely look at the screen. For each participant, we collected the fixation percentage (calculated as the sum of fixation durations on a particular AOI divided by the total time of stimuli exposure, which was 30 s per each condition). Additionally, the average fixation duration and the time to the first fixation on the AOI were examined.

A Baby Sensitivity Index (BSI) was calculated as the difference between the fixation percentage on all cute stimuli (*babies*,* animals*) and the fixation percentage on all non-cute stimuli (*adults, neutral objects*). A high score indicated a preference towards cute stimuli and a low score indicated a preference towards non-cute stimuli. Second, a Human Baby Sensitivity Index (Human BSI) was calculated as the difference between the fixation percentage on *babies* and the fixation percentage on *adults*. A high score indicated a preference towards *babies* and a low score indicated a preference towards *adults*.

### Data Analysis

First, to investigate the differences between groups related to each AOI from each condition, repeated measure MANOVAs and post-hoc t-test analyses were run in IBM Statistics version 26. Based on our hypotheses and study design, significant three-way interactions were expected to be driven by greater visual biases of the TD and LMS ASD groups towards cute stimuli within and between conditions, compared to the HS ASD group. The 3 × 2 × 3 repeated measure MANOVAs were separately run on each of the eye-tracking parameters: fixation percentage, average fixation duration, and time to first fixation. Each MANOVA was conducted using a 3-level between-group variable (*group*: HS ASD, LMS ASD, and TD), a 2-level within-group variable (*Type of Frame*: Condition 1 - “Human Adult” and Condition 2 - “Human Baby”), and a 3-level within-group variable (*Stimuli*: humans, animals, neutral objects). Wilks’s lambda *F*-test statistics are further reported. The post-hoc independent samples t-tests looked into differences between groups on each stimulus type. The paired samples t-tests were run to identify differences within group on the following relevant comparisons: *adults* versus *babies* (between conditions), *adults *versus *animals* (condition 1), *adults* versus *neutral objects* (condition 1), *animals* versus *neutral objects* (condition 1), *babies* versus *animals* (condition 2), *babies* versus *neutral objects* (condition 2), *animals* versus *neutral objects* (condition 2). To control for false discovery rates (FDR) in multiple comparisons, we have applied Benjamini and Hochberg’s procedure (1995) to control for the proportion of false discoveries using the MATLAB function *fdr_bh.m* (Groppe, 2015). The Benjamini and Hochberg’s FDR correction procedure adjusted the *p*-value threshold at *p* < .012. The visual graphs were created in Graph Pad Prism, version 8.4.3. To examine the association between BSIs and symptom severity (social affect and RRB), two separated regression analyses were conducted and illustrated graphically using R Studio 1.4.1103.

## Results

### Fixation Percentage

The 3 × 2 × 3 repeated measure MANOVA showed a significant three-way interaction (*F*(4,180) = 3.68, *p* = .007, partial η^2^ = .08). The interaction remained significant when age was introduced as covariate (*F*(4,178) = 3.34, *p* = .012, partial η^2^ = .07). A total of 39 comparisons (independent and paired samples t-tests) in which we expected differences were run.

Next, between-group post-hoc t-tests revealed that the HS ASD group spent significantly more time on *adults* (condition 1) than the LMS ASD group (*t*(61) = 2.87, *p* = .006), a result which remained significant after applying the Benjamini and Hochberg’s correction procedure.

Within-group post-hoc t-tests showed that LMS ASD and TD groups spent more gaze time on stimuli depicting *babies* than those depicting *adults* (*t*(39) = 2.51, *p* = .016, respectively *t*(30) = 2.12, *p* = .042), whereas the HS ASD group spent more time on *adults* than *babies* (*t*(22) = -2.31, *p* = .031). However, after applying the Benjamini and Hochberg’s correction procedure, none of these comparisons reached the significance threshold.

In condition 1, the TD group and the HS ASD group spent significantly more time on *adults* rather than *neutral* stimuli (*t*(30) = 3.36, *p* = .002, respectively, *t*(22) = 4.35, *p* < .001), which was not significant in the LMS ASD group (*t*(39) = 1.98, *p* = .055). However, the TD group and the LMS ASD spent significantly more time looking at *animals* rather than *neutral* stimuli (*t*(30) = 3.80, *p* = .001 and *t*(39) = 3.45, *p* = .001, respectively). The significant results were maintained after applying the Benjamini and Hochberg’s corrected threshold. In the HS ASD, the initial result indicating that the group spent significantly more time on *animals* than *neutral* stimuli (*t*(22) = 2.31, *p* = .031) did not survive the corrected threshold.

**Fig. 2:**
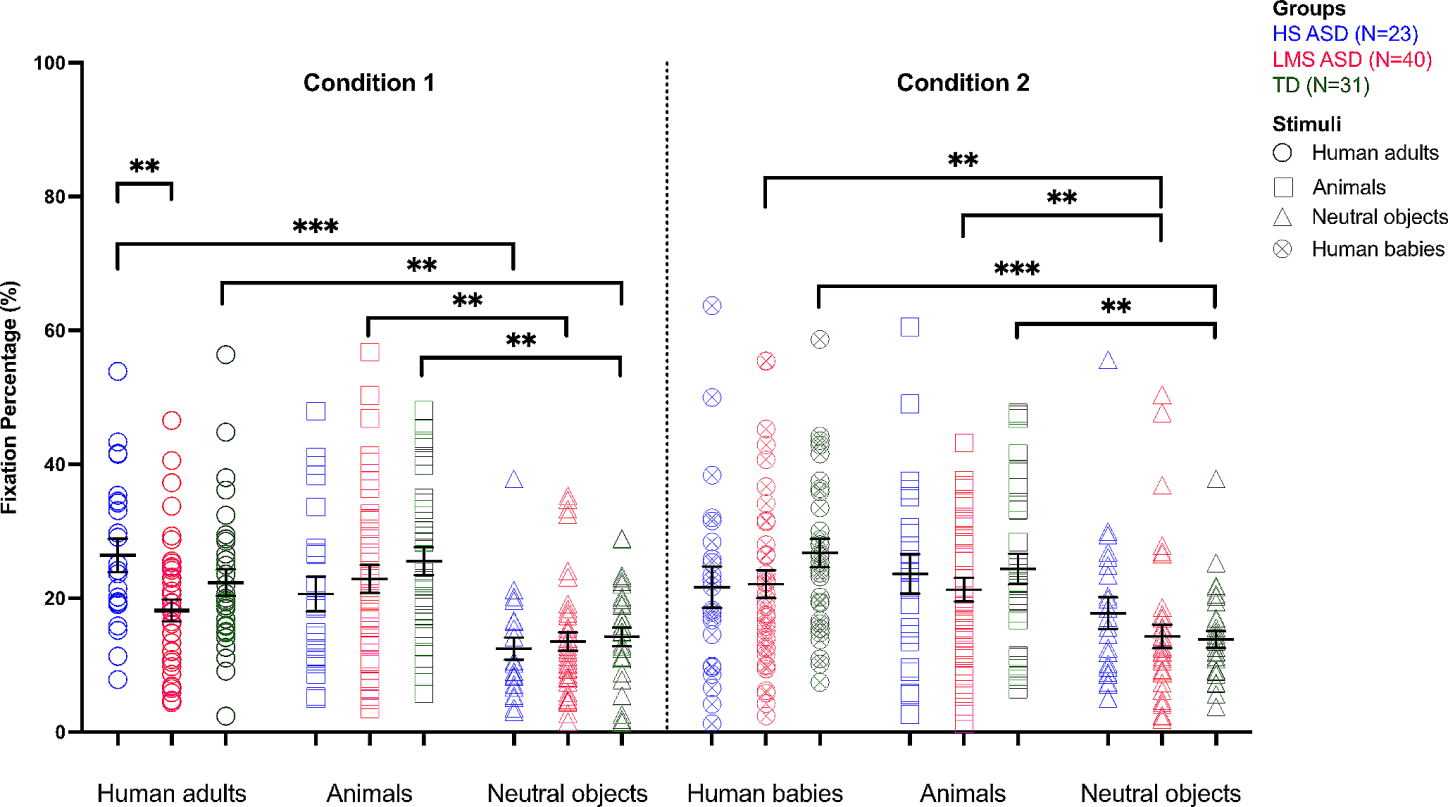
Mean scores of fixation percentage for each group, across conditions and stimuli. Error bars represent standard errors.* Note*: **p < .02; ***p < .001

In condition 2, the LMS ASD group spent significantly more time on *babies* and *animals* compared to *neutral* stimuli (*t*(39) = 2.70, *p* = .010, respectively, *t*(39) = 2.68, *p* = .011). The same pattern was found in the TD group (*t*(30) = 4.64, *p* < .001 and *t*(30) = 3.72, *p* = .001, respectively). These results were also maintained after applying the Benjamini and Hochberg’s corrected threshold. Within-group differences were not reproduced in the HS ASD group (*t*(22) = 0.88, *p* > .05, and *t*(22) = 1.39, *p* > .05 respectively). See Fig. 2; Table 3.

**Table 3:** Means and standard deviations of the fixation percentage on each AOI, in each condition, across the three groups

	Groups		
	HS ASD	LMS ASD	TD
AOIs in Condition 1			
Human adults	26.42 (11.98)	18.21 (10.30)	22.34 (11.03)
Animals	20.67 (12.43)	22.90 (13.14)	25.56 (11.65)
Neutral objects	12.49 (7.82)	13.54 (8.64)	14.22 (7.61)
AOIs in Condition 2			
Human babies	21.67 (14.77)	22.13 (13.14)	26.77 (11.79)
Animals	23.66 (14.10)	21.30 (11.14)	24.39 (12.67)
Neutral objects	17.79 (11.42)	14.31 (11.07)	13.87 (6.96)

*Note* AOI = area of interest

Additionally, to better understand the role of age for this particular significant result, Pearson correlations were run to explore potential associations between age and fixation percentages on each of the AOIs, in each group. Therefore, 18 Pearson correlations were run, setting a Bonferroni corrected *p*-value threshold of .002. The only significant correlation was a negative weak association between age and fixation percentage on stimuli depicting babies in the HS ASD (*r* = -.47, *p* = .025), which did not survive the Bonferroni correction.

### Average Fixation Duration

A 3 × 2 × 3 repeated measure MANOVA showed no significant three-way interaction, *F*(4,180) = 2.12, *p* = .081, partial η^2^ = .05.

### Time to First Fixation

A 3 × 2 × 3 repeated measure MANOVA showed no significant three-way interaction (*F*(4,180) = 0.47, *p* = .76, partial η^2^ = .01), but only a significant main effect of the stimuli (*F*(2,90) = 17.34, *p* < .001, partial η^2^ = .28). On average, across frames, the time to first fixation on stimuli depicting *humans* was shorter than compared to *animals* and *neutral objects*.

### Baby Sensitivity Index

**Fig. 3:**
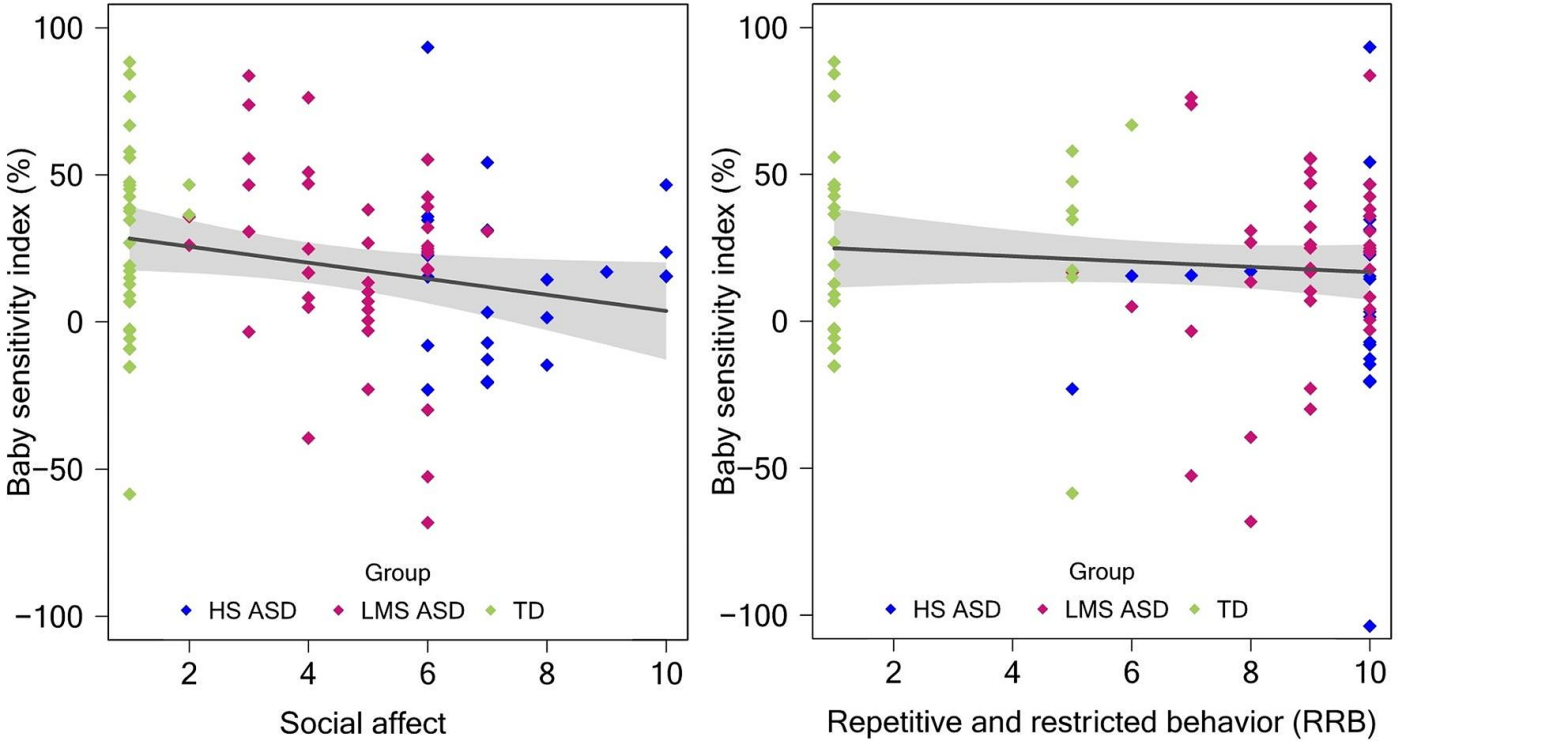
Correlations between overall baby sensitivity index (cute versus non-cute stimuli) and social affect and repetitive and restricted behavior calibrated severity scores based on the ADOS across the entire sample (*N* = 94). *Note*: X-axis origin = 1. While the groups are color-coded in the graph, the grouping variable was not included the regression model

First, social affect significantly predicted the BSI in the entire sample: *B* = -2.74, β = -.22, *t*(91) = -2.10, *p* = .04. Social affect scores explained a small proportion of variance in BSI: adjusted *R*^2^ = .04. When age was introduced in the regression model, the model was still significant (*p* = .04, adjusted *R*^2^ = .05) and social affect remained the unique significant predictor: *B* = -3.19, β = -.25, *t*(90) = -2.39, *p* = .02. With the HS ASD group removed from the sample, social affect is no longer a significant predictor of BSI: *B* = -2.95, β = -.19, *t*(68) = -1.56, *p* = .12; adjusted *R*^2^ = .02). With the TD group removed from the sample, the social affect is not anymore predicting the BSI (*B* = -3.97, β = -.22, *t*(61) = -1.75, *p* = .09; adjusted *R*^2^ = .03) in autistic participants (HS and LMS ASD groups combined). RRB did not significantly predict BSI: *B* = -.91, β = -.10, *t*(91) = -0.92, *p* = .36; adjusted *R*^2^ = -.002). When the three groups were taken separately, a significant result was found only within the LMS ASD group in relationship with social affect (*p* = .048). See Fig. 3.

**Fig. 4:**
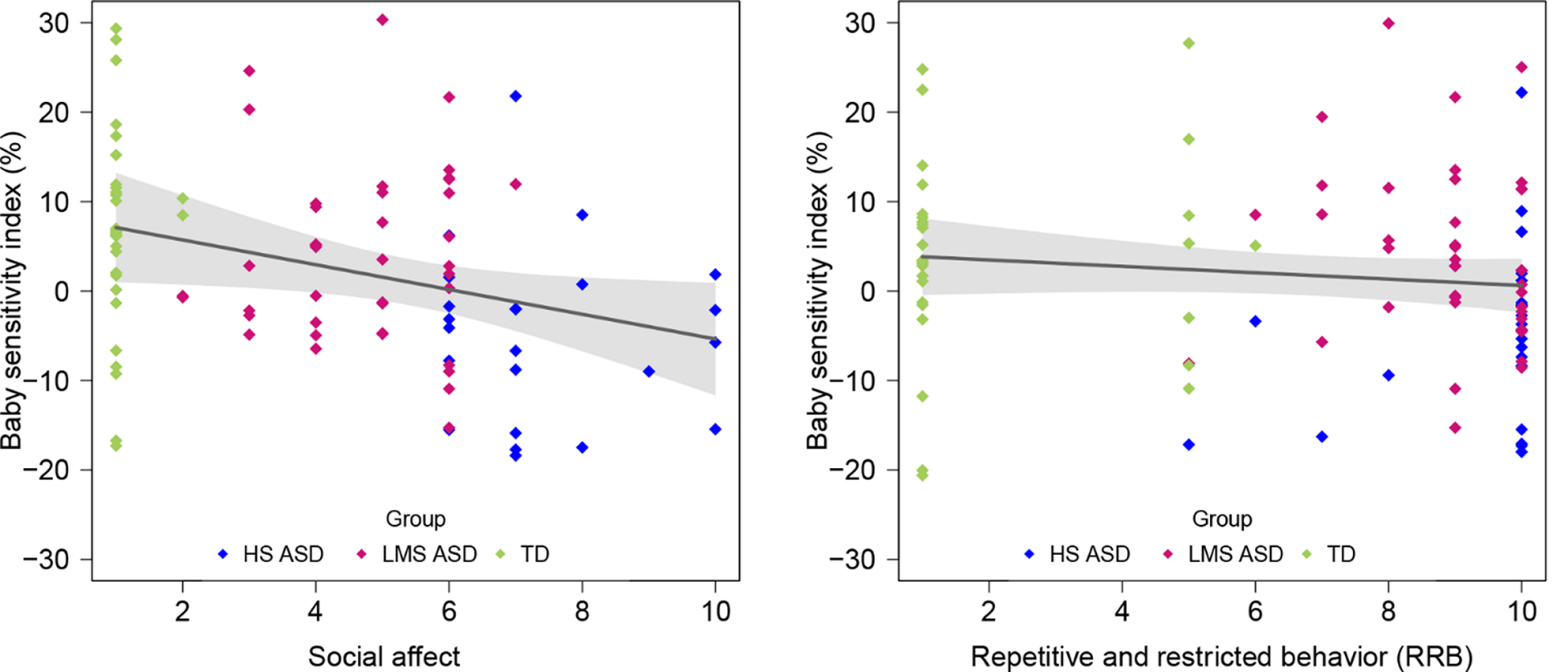
Correlations between human baby sensitivity index and social affect and repetitive and restricted behavior calibrated severity scores based on the ADOS across the entire sample (*N* = 94). *Note*: X-axis origin = 1. While the groups are color-coded in the graph, the grouping variable was not included the regression model

Second, social affect significantly also predicted the human BSI in the entire sample: *B* = -.97, β = -.24, *t*(91) = -2.32, *p* = .02. Social affect scores explained a small proportion of variance in BSI: adjusted *R*^2^ = .05. When age was introduced in the regression model, social affect remained the unique significant predictor: *B* = -.94, β = -.23, *t*(90) = -2.19, *p* = .03; adjusted *R*^2^ = .04. With the HS ASD group removed from the sample, social affect is no longer a significant predictor of BSI (*B* = .10, β = .2, *t*(68) = 0.17, *p* = .87). With the TD group removed from the sample, the social affect remained a significant predictor of BSI (*B* = -1.58, β = -.28, *t*(61) = -2.28, *p* = .03; adjusted *R*^2^ = .06) in autistic participants (HS and LMS ASD groups combined). RRB did not significantly predict BSI: *B* = -.36, β = -.12, *t*(91) = -1.13, *p* = .26; adjusted *R*^2^ = .003. See Fig. 4. When the three groups were taken separately, no significant results were found within each group (*p* > .05). As for the overall BSI, these changes occurring when one group is removed or when they are analyzed separately are likely because of the low variance (See Figs. 3 and 4 in this manuscript, and Fig. S4 in the supplementary material).

## Discussion

The current study examined whether autism symptom severity in young children is associated with visual exploration of cute social stimuli using a novel eye-tracking task. The task triggered differential visual patterns in relation to the heterogeneity of ASD profiles, supporting the potential of eye-tracking to study autistic traits (Mastergeorge et al., 2021).

As expected, comparisons following the three-way significant interaction suggest that only children with lower autistic symptoms (LMS ASD) and typically developing (TD) children spent significantly more time on cute stimuli than neutral objects. LMS ASD and TD groups looked more at *animals* than at *neutral objects* (condition 1: *adults*-*animals*-*neutral*), and more at *babies* and *animals* than at *neutral objects* (condition 2: *babies*-*animals*-*neutral*). Although TD participants showed a substantial visual bias towards cute stimuli, they looked significantly more at *adults* than *neutral objects* in condition 1, similar to HS ASD group.

Next, no significant interactions were found regarding children’s engagement with stimuli (average fixation duration), nor their initial fixations. All children oriented their initial fixations to stimuli depicting humans significantly faster than to the other stimuli. This result adds to the contradictory evidence about initial orientations in autism (Wang et al., 2020; Wilson et al., 2010). Given that differences in brain network specialization may occur with age (Johnson, 2014), longitudinal studies could examine whether groups gaps may latterly appear.

Finally, the findings indicate a significant correlation between baby sensitivity indexes and autism symptom severity in social affect, but not repetitive and restricted behaviors: the more social difficulties children had, the less fixation percentage they allocated to *cute stimuli* compared to *non-cute stimuli*, and to *babies* compared to *adults*, respectively.

Overall, our results indicate that autistic symptoms account for the visual patterns toward cute stimuli. Consistent with previous studies (Franchini et al., 2017; Zagury-Orly et al., 2022), social visual exploration in autistic children seems related to symptom severity, particularly to social affect. Social difficulties thus impact not only the preference towards robots (Kumazaki et al., 2017) and face processing (Zagury-Orly et al., 2022) but also visual attention towards cute-featured stimuli. Reduced attraction to baby schema in children with higher autism symptom severity may be negatively linked to affiliative interactions, such as play (e.g., fewer shared experiences, atypical toy play, increased solitary play) and impact, consequently, their socio-emotional development (Elbeltagi et al., 2023; Golonka et al., 2023; Zaharia et al., 2022). Indeed, more social difficulties with peers are reported in children with high autistic levels (Sari et al., 2021). Furthermore, in contrast to previous findings (Grandgeorge et al., 2016), no significant preference for *animals* over *humans* was revealed in autistic children. Thus, it may be worthwhile to consider these findings for the design of agents used in interventions for autistic children (e.g., dolls, virtual agents, robots; e.g., Stallmann et al., 2022; Yao et al., 2022) and their implications for occupational, play, and animal-assisted therapies. Considering the unexpected lack of a significant preferential distinction between *adults* (non-cute) and *animals* (cute) in Condition 1 within both LMS ASD and TD groups, this finding contributes to the mixed results found in the literature. Descriptively, TD and LMS ASD groups show a higher fixation percentage on *animals* than *adults*, but the difference does not reach statistical significance. We posit that stimuli featuring *adults* serve as significant competitors to cute-featured stimuli, and their potential relevance to participants lies in the depiction of caregiving figures that are crucial for one’s survival and response to primary needs.

Given that relevance typically drives attention to baby faces (Brosch et al., 2007, 2008; Pool et al., 2016), these visual patterns may also be explained by group differences in the appraisal of cute stimuli. As alterations in the perception of relevant social targets (Chawarska et al., 2016) and in the appraisal of negative emotions (Sharma et al., 2014) may occur in autistic individuals, our findings suggest that autistic symptom levels could impact the appraised relevance. Considering that little is known about appraisal in atypical development and that children’s emotional experience might differ from adult research (Walle & Özden, 2024), the present study may suggest that cute stimuli have lower relevance for the children in the HS ASD group. Eventually, our findings may be partially explained by alterations in social reward processing in autism, which are also expected to depend on the symptom severity (Bottini, 2018).

Furthermore, it has been claimed that the baby schema does not only induce the classical positive cuteness effect but also signals vulnerability and approachability (Sanefuji et al., 2007). This could induce an overarousal in certain autistic individuals, provoking personal distress and willingness to divert attention (Hadjikhani et al., 2014; Sanefuji et al., 2007). Consequently, cute-featured stimuli may lead to avoidance, possibly explaining the decreased time spent on cute stimuli in the HS ASD group. Therefore, for individuals with severe autistic symptoms, cute objects may represent another set of positive stimuli triggering atypical visual patterns, altering valence perception and disrupting, in such, the pleasant emotional experience (Antezana et al., 2022; Jacques et al., 2022; Zaharia et al., 2021).

### Limitations and Future Perspectives

Several limitations should be considered. First, the familiarity and previous exposure to the variety of cute-featured stimuli that children encounter throughout development (e.g., younger, or same-age siblings or peers, animals, dolls, cartoons; Damon et al., 2021; Saxton et al., 2020) may intertwine with social skills and attention. Although it is suggested that having siblings can increase the cuteness response in TD participants (Luo et al., 2015; Yao et al., 2022), a precise assessment of all the environmental elements potentially impacting their cuteness responses may be unattainable. Second, it is important to conduct such studies considering diverse cultural and socio-economic backgrounds in both autistic and TD groups (see Wood de Wilde et al., 2023). For instance, the “cute” culture (also known as “kawaii”) and anthropomorphism are widespread in Japan and being frequently exposed to them could lead to different visual preferences and patterns in the exploration of cute versus non-cute, and/or human versus non-human stimuli (Atherton et al., 2023). Next, although age was not found as a moderator in our analyses, future research should investigate these observations in age-matched groups to exclude any potential age-related variability in the gaze patterns (Dalrymple et al., 2018). Also, extending future research on cuteness in children above 6 years old, combined with their self-reported ratings, would help confirm the presence of a cuteness response and clarify the exact nature of differences between the groups. Moreover, the current study implemented a categorical approach to separate autistic participants into groups supported by ADOS-2 manual instructions and theoretical categorizations and the lack of density for some value ranges (see Fig. S4, supplementary material). Hence, the present data adds to the dimensional versus categorical debate around the nature of autism (Lefort-Besnard et al., 2020; Roberts et al., 2018). For instance, a recent brain study suggests the possibility of a reconciliation between these two approaches (Tang et al., 2020). For a more comprehensive understanding of heterogeneity, future studies should incorporate larger samples of autistic individuals. Another limitation is that the cuteness conceptualization used in this study is limited to only one facet of a multidimensional model (Doebel et al., 2022). Hence, the processing of other cute attributes (e.g., sounds, behavior; Golonka et al., 2023; Kringelbach et al., 2016) in autistic children should be further investigated. Worthy to note, although evaluated as cute, the age of animals was not controlled in the current study. To better understand these results and the generalization of baby schema sensitivity across species, it would be recommended to test the visual exploration of human babies versus human adults while presenting the stimuli simultaneously, as well as of cute animals versus non-cute animals by manipulating their physical traits (see Borgi et al., 2014). Further, responses to baby schema may depend on the stimuli characteristics (i.e., static, dynamic, real-world) (Chevallier et al., 2015; Klin et al., 2002; Mouga et al., 2021), rendering generalizations difficult. For example, a tactile interface allowing interaction with stimuli (Lio et al., 2019) might increase the ecological relevance of the findings and the robustness of eye-tracking measures. Using multi-method approaches to examine simultaneously attentional processes and the subjective experience towards cute stimuli may provide additional insight. Finally, an important methodological limitation refers to the Tobii Studio software which provides only a qualitative visual verification of the calibration accuracy and precision. Using software providing a quantitative procedure to evaluate the eye-tracking metrics quality for each participant would improve the validity of the findings and conclusions (Dalrymple et al., 2018).

## Conclusion

The current study provides evidence for an altered attentional bias toward baby schema in children with high autism severity and suggests that the decreased attention to cute stimuli may be related to social difficulties. These findings might help better understand the appraised relevance, social reward processing, and emotional experience during the exploration of cute stimuli across the autism spectrum. Variations linked to the symptom severity observed in the cuteness responses may have important implications in considering individualized approaches in therapies or in the design of interactive agents used in interventions for autistic children. Future studies should examine the extent to which the emotional reward learning of baby schema (Kinard et al., 2020; Stussi et al., 2018) and the sensitivity to other cute features (Golonka et al., 2023) are affected in autism. Finally, it is worthy to examine how baby schema sensitivity concretely contributes to the unfolding of social interactions and prosocial behavior across development.

## Electronic supplementary material

Below is the link to the electronic supplementary material.

Supplementary Material 1

## Data Availability

The datasets analyzed during the current study are available in the Open Science Framework repository, DOI 10.17605/OSF.IO/CA8VU.
