# Supplementary material for: Examining the Link Between Social Affect and Visual Exploration of Cute Stimuli in Autistic Children
Source: J Autism Dev Disord. 2024 Aug 22;55(12):4212–25. doi: 10.1007/s10803-024-06504-1 (PMC12589387; doi:10.1007/s10803-024-06504-1)
Supplement: Supplementary file 1 — Supplementary Material 1 [file 10803_2024_6504_MOESM1_ESM.docx]

**Supplementary material**

**Additional data analysis**

Given the dimensional approach and the profile heterogeneity in autism (Lefort-Besnard et al., 2020; Roberts et al., 2018), additional multilevel ANOVA analyses were run on each of the eye-tracking parameters (fixation percentage, average fixation duration, time to first fixation) using the continuous ADOS CSS scores (total, RRB, and social affect) as predictors. This analysis was conducted to be contrasted with the analyses that used categorical autism groups.

First, multilevel ANOVAs were run for ADOS total CSS and, second, for RRB and social affect together, due to the high correlation between these two predictors (*r* = .75, *p* > .001). The additional analyses were run in R-Studio (R Core Team, 2022) using the following packages: *lme4* (Bates et al., 2015), *lmerTest* (Kuznetsova et al., 2017), *car* (Fox & Weisberg, 2019), *emmeans* (Lenth et al., 2024).

**Results**

The multilevel ANOVAs showed no significant three-way interactions on any of the ADOS scores: a) *Type of Frame × Stimulus × Total score* (fixation percentage: *F*(2, 558) = 2.20, *p* > .05; average fixation percentage: *F*(2, 465) = 0.72, *p* > .05; time to first fixation: *F*(2, 465) = 0.15, *p* > .05; see Fig. S1), b) *Type of Frame × Stimulus × Social Affect* (fixation percentage: *F*(2, 558) = 2.06, *p* > .05; average fixation percentage: *F*(2, 465) = 0.70, *p* > .05; time to first fixation: *F*(2, 465) = 2.32, *p* > .05; see Fig. S2), c) *Type of Frame × Stimulus × RRB* (fixation percentage: *F*(2, 558) = 0.38, *p* > .05; average fixation percentage: *F*(2, 465) = 0.20, *p* > .05; time to first fixation: *F*(2, 465) = 1.86, *p* > .05; see Fig. S3).

**Limitations**

The additional analyses confirmed that ultimately categorical autism groups were preferred for these data. Importantly, the density of the continuous trait variables showed evidence of bi- or multi-modality, suggesting that the ADOS CSS scores were not properly continuous but latent categorical (see Fig. S4), with some value ranges completely unobserved. Moreover, exploratory modelling suggested that some of the associations between continuous trait variables and the eye tracking outcomes were nonlinear (e.g., quadratic or cubic). Although this could have been incorporated into our multilevel analyses, even a quadratic model would be forced to interpolate the association for the missing value ranges. By contrast, the use of categorical groups avoids interpolation and leaves the pattern of means between trait and outcome unconstrained. Finally, although the empirical densities favored a categorical approach, we used theoretical cutoffs from the ADOS-2 manual to define the groups, not empirical ones. This option was preferred because empirical cutoffs are less stable, changing from sample to sample.

**References**

Bates, D., Mächler, M., Bolker, B. M., & Walker, S. C. (2015). Fitting linear mixed-effects models using lme4. *Journal of Statistical Software*, *67*(1), 1–48. https://doi.org/10.18637/jss.v067.i01

Fox, J., & Weisberg, S. (2019). *An {R} Companion to Applied Regression* (3rd ed.). SAGE.

Kuznetsova, A., Brockhoff, P. B., & Christensen, R. H. B. (2017). lmerTest Package: Tests in Linear Mixed Effects Models. *Journal of Statistical Software*, *82*(13), 1–26. https://doi.org/10.18637/JSS.V082.I13

Lefort-Besnard, J., Vogeley, K., Schilbach, L., Varoquaux, G., Thirion, B., Dumas, G., & Bzdok, D. (2020). Patterns of autism symptoms: hidden structure in the ADOS and ADI-R instruments. *Translational Psychiatry*, *10*(257), 1–12. https://doi.org/10.1038/s41398-020-00946-8

Lenth, R. V., Bolker, B., Buerkner, P., Gine-Vazquez, I., Herve, M., Jung, M., Love, J., Miguez, F., Riebl, H., & Singmann, H. (2024). Estimated Marginal Means, aka Least-Squares Means. In *American Statistician*. https://github.com/rvlenth/emmeans

R Core Team. (2022). *R: A language and environment for statistical computing. R Foundation for Statistical Computing*. https://www.r-project.org

Roberts, J. E., Ezell, J. E., Fairchild, A. J., Klusek, J., Thurman, A. J., McDuffie, A., & Abbeduto, L. (2018). Biobehavioral composite of social aspects of anxiety in young adults with fragile X syndrome contrasted to autism spectrum disorder. *American Journal of Medical Genetics, Part B: Neuropsychiatric Genetics*, *177*(7), 665–675. https://doi.org/10.1002/ajmg.b.32674

**Fig. S1** Graph illustrating the results of the multilevel ANOVA analyses on the ADOS total CSS as a continuous variable across *type of frame* (Condition 1 - “Human Adult” and Condition 2 - “Human Baby”) and *stimuli* (humans, animals, neutral objects) in the entire sample (N = 94)

**Fig. S2** Graph illustrating the results of the multilevel ANOVA analyses on the social affect CSS as a continuous variable across *type of frame* (Condition 1 - “Human Adult” and Condition 2 - “Human Baby”) and *stimuli* (humans, animals, neutral objects) in the entire sample (N = 94)

**Fig. S3** Graph illustrating the results of the multilevel ANOVA analyses on the RRB CSS as a continuous variable across *type of frame* (Condition 1 - “Human Adult” and Condition 2 - “Human Baby”) and *stimuli* (humans, animals, neutral objects) in the entire sample (N = 94)

**Fig. S4** Graph illustrating the distribution of the ADOS CSS scores: total, RRB, and social affect across all participants (N = 94): typically developing children (ADOS total CSS score range: 1 to 2), low-to-moderate severity ASD group (ADOS total CSS score range: 4 to 7), and high severity ASD group (ADOS total CSS score range: 8 to 10)
